# Educational Gradient of Multi-partner Fertility: First Estimates for the UK

**DOI:** 10.1007/s10680-024-09708-4

**Published:** 2024-06-26

**Authors:** Sebastian Stannard, Ann Berrington, Nisreen A. Alwan

**Affiliations:** 1 Department of Social Statistics and Demography, University of Southampton, Southampton, UK; 2 ESRC Centre for Population Change, University of Southampton, Southampton, UK; 3 School of Primary Care, Population Sciences and Medical Education, Faculty of Medicine, University of Southampton, Southampton, UK; 4 University Hospital Southampton, NHS Foundation Trust, Southampton, UK

**Keywords:** Multi-partner fertility, Partnerships, Childbearing, Family formation, Education

## Abstract

Recent demographic changes in Western countries have resulted in higher rates of partnership dissolution and serial partnering, and an increase in childbearing across multiple partnerships. This has given rise to more complex family dynamics including multi-partner fertility (MPF), defined as having biological children with two or more partners. Yet estimates of MPF in the UK have not previously been available. This paper describes an ‘indirect approach’ to estimate the prevalence of MPF in the UK, for men and women, given different assumptions. The paper additionally explores differences in MPF according to own and parental educational attainment. Amongst those born in Britain in 1970, 12–14% of men and 15–18% of women experienced MPF by age 42, depending on the assumptions made. For most of the cohort, MPF occurred with two different coresidential partners. We have established that MPF is a common family formation in the UK, but there are large educational disparities in MPF prevalence.

**Supplementary Information:**

The online version contains supplementary material available at 10.1007/s10680-024-09708-4.

## Introduction

Family dynamics in Western countries have become increasingly unstable and complex including increased partnership dissolution, re-partnering and childbearing across multiple partnerships. Multi-partner fertility (MPF) is defined as an individual having biological children with two or more partners (Carlson & Furstenberg, 2006; Guzzo, 2014; Petren, 2017). In the late eighteenth and nineteenth centuries it was not uncommon for a widowed parent to remarry and have additional children. Nor was it uncommon during the mid-twentieth century for young unmarried women who became pregnant to put their child up for adoption, then later form a marriage with a different man and have additional children (Guzzo, 2014). Today, however, MPF is common amongst those who have a child with one partner and then separate, subsequently forming another partnership and then having additional children.

It is important that research assesses the prevalence and distribution of MPF given parents and children who experience MPF may face disruption and uncertainty across several domains including daily routines, geographical location, custody arrangements, entering and leaving single parenthood and losses of economic and social capital (Dorius, 2010; Petren, 2017). Subsequently, MPF can result in poorer socioeconomic and health outcomes (Carlson, 2006; McLanahan, 2004; Monte, 2019). However, it may also be the case that worse outcomes are due to the selection of those from poorer socioeconomic and educational backgrounds into behaviours indicative of MPF such as an earlier age at parenthood, non-marital childbearing, and higher rates of separation and re-partnering (Andersson, 2021; Jalovaara et al., 2022; Martin, 2006; McLanahan, 2004). Parental background factors including social class, education and partnership dissolution, and childhood experiences such as physical health, cognition and psychological attributes are associated with the family dynamics that are structurally linked to MPF including early childbearing, high parity, partnership separation and serial partnering (Berrington & Diamond, 2000; Carr & Springer, 2010; Holley et al., 2006; Schoen, 2020; Shearer et al., 2002). McLanahan’s (2004) ‘diverging destinies’ suggested that educational attainment is one of the key drivers behind differing non-traditional family behaviours (delayed marriage, cohabitation, non-marital childbearing, and divorce). Therefore, it is important to understand if ‘diverging destinies’ extends beyond the family behaviours previously identified to more complex family dynamics such as MPF, and document both the scale of MPF and examine the socioeconomic differences of those who have experienced MPF.

No previous estimates of MPF for the UK have been made, so we are not able to provide a description of historical trends in the prevalence of MPF in the UK. However, we can evaluate historical trends in the demographic factors associated with MPF. In the UK, the delay in age at entry into parenthood, decreased family sizes and increased proportions who remain childless might lead us to expect a reduction in the level of MPF over historical time. Completed family size fell from 2.07 children per woman for those born in 1950 to 1.91 for women born in 1970, whilst the proportion remaining childless increased from 14% for women born in 1950 to 17% for those born in 1970 (ONS, 2022). However, rapid changes in partnership dynamics over the same period have offset these factors to potentially cause an elevated chance of MPF in the UK.

The liberalisation of divorce laws and changing attitudes and norms relating to marriage and cohabitation were associated with sharp rises in divorce rates, declines in first marriage rates and increased cohabitation from the 1970s onwards (Kalmijn, 2020; Perelli-Harris et al., 2017). Most UK couples now choose to cohabit before marriage, but only some cohabitors progress to marriage. Cohabiting couples are increasingly likely to separate prior to marriage (Boertien, 2020). Given these high levels of partnership dissolution and serial partnering (Bukodi, 2012), it seems likely that the UK will have experienced an increase in the level of MPF.

There are also several demographic reasons why we might expect a high prevalence rate in the UK, comparable to, or higher than, the MPF rates found in previous studies conducted in other Western countries. Teenage birth rates, whilst having declined, remain higher in the UK than many other Western countries (Sedgh et al., 2015), and a substantial proportion of children are born outside of any coresidential union (ONS, 2013). Amongst parents, a relatively high proportion go on to have third and fourth births (Eurostat, 2023), and levels of partnership dissolution (either divorce or cohabitation separation) are high in the UK compared to many other European countries (Coleman, 2013) with almost a quarter of families with dependent children headed up by a single parent (ONS, 2022). Given these differences in demographic trends, it is important to explore if the prevalence of MPF is especially high in the UK.

## Existing Literature

Academic interest in MPF has developed rapidly over the past two decades. American scholars led the way, using data from the Fragile Families and Child Wellbeing Study to provide one of the first estimates of MPF (Carlson & Furstenberg, 2006). Subsequently, researchers in the US and Europe provided further estimates, as indicated by Table 1, that includes a demonstrative list of published estimates of MPF found through a scoping literature search. These do not include estimates from unpublished work or from papers not directly focussed on MPF. Calculations based on adults of all ages (top panel) are, not surprisingly, lower (generally under 10%), than for those calculations based on adults who have reached midlife (15–27%) (bottom panel).

**Table 1:** Published MPF estimates, separated into estimates based on adults of all ages and adults aged in their forties, and ordered according to country and publication year

Author	Data source	Country	Method	MPF prevalence
*Estimates based on adults of all ages*				
Carlson and Furstenberg (2006)	Fragile Families and Child Wellbeing Study (now the Future of Families and Child Wellbeing study)	USA	Mothers were asked to report whether she or the child father had any other children with a different partner	**25%** of parents reported a child with multiple partners^a^
Guzzo and Furstenberg (2007a)	National Survey of Family Growth	USA	Information on men’s fertility experiences situated within relationships e.g., men were asked if they had any children with each partner	**8%** of men aged 15–44
Guzzo and Furstenberg (2007b)	National Longitudinal Survey of Adolescence Health	USA	Fertility information collected in reference to specific relationship(s) e.g., for each birth partner information was available	**11%** of women aged 15 and older
Manlove et al. (2008)	National Survey of Family Growth	USA	Information on fathers’ fertility experiences situated within relationships e.g., fathers were asked if they had any children with each partner	**17%** of fathers aged 16–45
Evenhouse and Reilly (2010)	Survey of Income and Programme Participation	USA	Derived from household relationship matrices	**10%** of women aged 15 and older
Monte (2017)	Survey of Income and Programme Participation	USA	Direct question included in the survey	**8.6%** of men aged 15 and older
				**11.4%** of women aged 15 and older
Thomson et al. (2020)	US Survey of Family Growth	USA	MPF was derived using an indirect approach where each birth was situated within respondents’ partnership histories, using both the dates of start and end of unions and the date of birth of biological children. Percentage is based on portion of total fertility	**21%** of women the USA aged 15–49
Fostik and Bourdais (2020)	General Social Survey	Canada	MPF was derived using an indirect approach where each birth/adoption was situated within respondents’ conjugal trajectory, using both the dates of start and end of unions and the date of birth of biological children and the date of arrival of adopted children	**5.3%** of men aged 25–64
				**7.5%** of women aged 25–64
Lappegård and Rønsen (2011)	National register data for Norwegian population	Norway	Linked birth of child to biological father and the mothers to determine if each birth was with the same or a new partner	**4%** of men in cohorts born before 1938
				**11%** of men in cohorts born in early 1960s
Grey and Evans (2008)	HILDA survey	Australia	Identified respondents who had children across more than one marriage, and those who had their children outside of marriage	**10%–17%** of men aged 15 and over
				**13%–20%** of women aged 15 and over
Thomson et al. (2020)	Generations and Gender Survey and Harmonised Histories	Europe	MPF was derived using an indirect approach where each birth was situated within respondents’ partnership histories, using both the dates of start and end of unions and the date of birth of biological children. Percentages are based on portion of total fertility	**3%** of women in Georgia aged 18–80
				**5%** of women in Bulgaria aged 18–80
				**4%** of women in Spain aged 15–98
				**5%** of women in Romania aged 18–80
				**7%** of women in Lithuania aged 18–79
				**7%** of women in Hungary aged 21–79
				**6%** of women in Poland aged 18–84
				**8%** of women in Belgium aged 18–80
				**10%** of women Czech Republic aged 18–79
				**9%** of women in France aged 18–79
				**9%** of women in Sweden aged 18–80
				**9%** of women in Norway aged 19–81
				**11%** of woman in Austria aged 18–46
				**12%** of women in Estonia aged 21–81
*Estimates based on adults aged in their forties*				
Dorius (2010)	National Longitudinal Survey of Youth 1979	USA	Comparing union and fertility experiences within household composition	**18.8%** of women aged 41–49
Guzzo (2014)	National Longitudinal Study of Adolescent Health	USA	Fertility information collected in reference to specific relationship e.g., for each birth partner information was available	**13%** of men aged 40–44
				**19%** of women aged 41–49
Jalovaara and Kreyenfeld (2020)	Statistics Finland—longitudinal population registers	Finland	Estimated by comparing the date and year of each birth and the ‘id’ codes of the registered parent	**15%** of men mean aged 42
				**19%** of women mean aged 42
Jalovaara and Kreyenfeld (2020)	German Family Panel—pairfam;	Germany	Estimated from respondents reporting the partnership a child is born into	**10%** of men in West Germany mean aged 40.3
				**16%** of women in West Germany mean aged 40.5
				**19%** of men in East Germany mean aged 41.2
				**27%** of women in East Germany mean aged 42

^*a*^*Estimates may be higher because the paper only considers those who were parents, and because the dataset used contained an oversample of non-marital birth*

In the USA, MPF prevalence varied between 8 and 9% for men and 10 and 11% for women (Evenhouse & Reilly, 2010; Guzzo & Furstenberg, 2007a, 2007b; Monte, 2017). Amongst men and women who were aged in their forties, MPF prevalence varied from 13 to 17% (men) and 11 to 20% (women) (Dorius, 2010; Guzzo, 2014; Manlove et al., 2008; Thomson et al., 2020). Compared to the USA, MPF in Canada was estimated to be much lower; 5% of men and 8% of women aged 25–64 (Fostik & Bourdais, 2020).

Across Europe MPF prevalence was more varied with lower prevalence reported in Norway (11%) (Lappegård & Rønsen, 2011; Lappegård & Thomson, 2018) and West Germany (10% of men; 16% of women) (Jalovaara & Kreyenfeld, 2020) compared to Finland (15% of men; 19% of women) (Jalovaara & Kreyenfeld, 2020), Sweden (22%) (Lappegård & Thomson, 2018) and East Germany (19% of men; 27% of women) (Jalovaara & Kreyenfeld, 2020). Thomson et al., (2020) estimated MPF via birth rate among women at risk of having a child with a new partner for 14 European countries. Their estimates ranged from 3 to 4% in Georgia and Spain respectively, to 11% in Austria and 12% in Estonia.

At a country level the prevalence of MPF is influenced by the level of fertility (Schoen, 2020) and partnership dynamics. For example, Lappegård and Thomson (2018) suggested the higher prevalence in Sweden as compared to Norway results from Sweden having a longer history of births in cohabitation, union dissolution and re-partnering. Additionally, MPF is influenced by contextual factors such as attitudes towards divorce, child custody arrangements, welfare support for lone parents and cultural norms regulating post-separation behaviour (Andersson, 2021). It is important therefore that MPF continue to be estimated for an array of countries.

The prevalence of MPF often varies between studies due to differing types of data sources utilised, the measurement approach used, and the populations studied. Some estimates are derived from household grids that focus on parental reports of children currently living within the household and therefore exclude any children outside the household (examples include Andersson, 2021; Evenhouse & Reilly, 2010; Grey & Evans, 2008; Meyer et al., 2005). Some scholars analyse the full population, whilst others focus on parents with one or more children, or parents with two or more children (the population at risk of having experienced MPF) (for examples see Andersson, 2021; Jalovaara & Kreyenfeld, 2020; Schoen, 2020; Thomson et al., 2020). Moreover, some scholars present estimates of MPF for limited age ranges, or for certain subgroups such as women or unmarried parents (for examples see Dorius, 2010; Guzzo & Furstenberg, 2007b; Thomson et al., 2020).

Monte and Fields (2020) highlight the need to consider men and women separately given there remains inadequate estimates of the prevalence of MPF for men partly because fathers are sometimes omitted from surveys either because they are not able to be identified or because there are errors in the reporting of paternity status (Rendall et al., 1999). The underreporting of men’s paternity status has been attributed to the fact that women physically bear children and that child rearing has historically been considered the domain of women, with some men not involved in their children’s lives and therefore fail to report a child (Forste, 2002). Additionally, men and women will likely be impacted differently by exposure to MPF, in part because fathers are less likely to retain custody of children (Monte & Fields, 2020). For these reasons, it is important to also explore gender differences in the prevalence of MPF.

This paper is the first to estimate MPF prevalence in the UK, this is an important addition to current family and fertility literature given MPF may be especially prevalent in the UK as discussed above. Additionally, we are able to provide those estimates for both men and women as both were asked full coresidential partnership and fertility histories. The 1970 British Cohort study (BCS70) has collected detailed information about past children living both within and outside the respondent’s household. Moreover, the prospective nature of the cohort study means that we can get more precise estimates of MPF up to age 42 because overlapping childbearing histories were collected at multiple time points across the cohort’s life course, thus reducing the likelihood of recall errors. This is especially important for men, who are reported to provide less accurate fertility histories (Rendall et al., 1999). Given direct information on MPF is unavailable in our dataset, our approach represents a method that allows for high quality datasets such as the BCS70 to be used. Finally, given concerns about the role of family instability and complexity on intergenerational transmission of disadvantage we examine overall descriptive trends in educational differences in MPF estimates.

## Estimating MPF Amongst the 1970 British Cohort Study

The 1970 British Cohort study (BCS70) has followed 17,196 participants born in Britain during a single week of 1970. Further details are reported elsewhere (Elliott & Shepherd, 2006). We utilise retrospective coresidential partnership and fertility histories collected at age 26, 30, 34, 38 and 42, which have been seamed together by the BCS70 survey team into a single history (University of London, 2016). Effort was made to repair erroneous cases where dates of birth and/or dates of coresidential partnership had been inconsistently recorded across sweeps and some of these decisions are outlined in Supplementary Materials 1. We completed some imputations for the 298 cases where coresidential partnership start months and/or coresidential partnership end months were not reported (Supplementary Materials 1).

MPF was estimated indirectly by comparing the reported month of birth of each biological child to the start and end dates of coresidential partnerships. An indirect approach should be used when no other suitable data is available, and the approach differs from the ideal direct approach where a question about MPF is asked directly to study participants.

We assumed that a birth was with a specific partner if the date of birth lay within the start and end dates of that coresidential partnership. When a birth occurred outside of a coresidential partnership, we made further assumptions about whether a birth reported closely before a partnership start date or shortly following a coresidential partnership dissolution involved the same partner (Stykes & Guzzo, 2019). Two assumptions were made. Firstly, those births occurring up to 6 months prior to a coresidential partnership formation were linked to this new coresidential partnership because non-coresidential relationships tend to either dissolve or transition to a coresidential relationship quickly following conception (Beaujouan & Bhrolchain, 2014; Lichter et al., 2014, 2016). Secondly, births up to 9 months following a coresidential partnership dissolution were linked with the previous partner as a child would have been conceived at the time of the previous union. These assumptions are the same as those made in Fostik and Bourdais (2020) estimates of MPF.

Next, we derived the total number of births within each reported coresidential partnership. We edited 34 cases where the same birth was counted in two separate coresidential partnership by assuming that the coresidential partnership that was closest to the birth date of the child was the coresidential partnership the child was born into.

Following that, we estimated the number of births that took place outside of a coresidential partnership and made further assumptions as to whether these births were all to the same parent, or different parents. The indirect method does not tell us who the other parent is, so we estimated a range: the maximum non-residential fertility assumed that all births outside of a coresidential partnership were with different partners, whilst the minimum non-residential fertility assumed that all births outside of a coresidential partnership were with the same partner.

The final step in estimating MPF prevalence involved combining the total number of births within each reported coresidential partnership with the minimum and maximum non-residential fertility. 86.8% of the cohort either had no children or all births were recorded within coresidential partnership(s), and 13.2% of all births took place outside of a coresidential partnership. The minimum non-residential fertility assumed that all 13.2% of births outside a coresidential partnership occurred with just one partner. The maximum non-residential fertility assumed that 8.7% of all births occurred with one partner outside a coresidential partnership, 2.9% occurred with two different partners, 1.0% with three different partners and 0.5% with four or more partners.

## Results

### MPF Prevelance

**Fig. 1:**
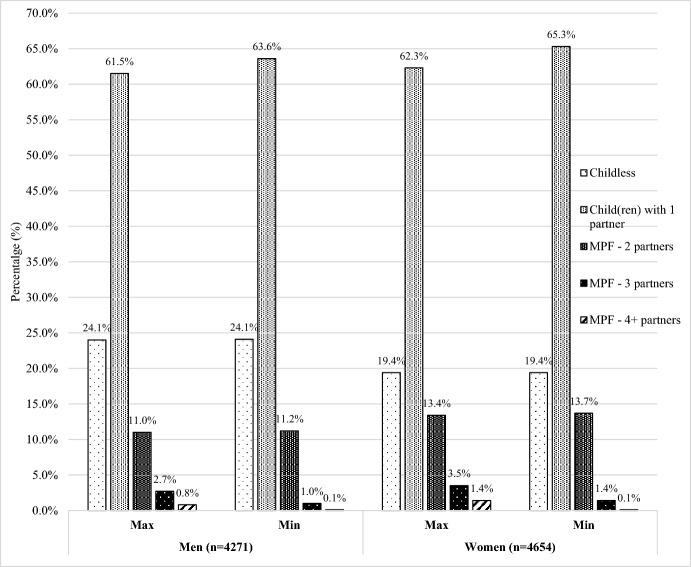
The percentage of men and women who have reported experiencing MPF, according to minimum and maximum assumptions regarding births outside of any coresidential partnership

As shown in Fig. 1, 12.2–14.4% of men and 15.2–18.3% of women born in 1970 experienced MPF by age 42, depending upon whether the minimum or maximum MPF for births outside any coresidential partnership is assumed. Assuming the maximum—that all births outside of a coresidential partnership were to different partners, 2.2% more men and 3.1% more women experienced MPF compared to assuming the minimum level of MPF. As shown in Fig. 1, the minimum and maximum MPF assumptions most strongly affect MPF estimates at higher levels of fertility partners (i.e., MPF with 3 or 4 or more partners) compared to the modal MPF (MPF with 2 partners). Figure 1 also indicates that, by age 42, men (24.0%) were more likely to report remaining childless compared to women (19.0%). However, men were less likely to report children with two partners (men 11.0–11.2%; women 13.4–13.7%), three partners (men 1.0–2.7%; women 1.4–3.5%) or four or more partners (men 0.4%; women 1.4%) compared to women.

**Fig. 2:**
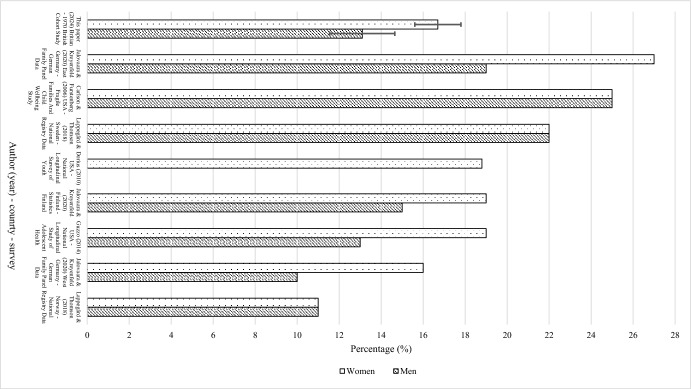
Distribution of MPF estimates compared to estimates in this paper, for studies that consider MPF based on adult in their forties

Figure 2 presents the UK estimates of MPF for men and women born in 1970 at age 42 compared to those for other countries for a similar age and historical period. The UK estimates are similar to those from the USA, Sweden, Finland, Norway and Germany. Our results are like that of Lappegård and Rønsen (2011) and Sobotka and Toulemon (2008), who suggested that amongst Norwegian and Danish populations 10–11% of people born in the early 1960s had experienced MPF by midlife. The prevalence is similar to that found by Dorius (2010) for the USA (19% of women aged 41–49 experienced MPF) and by Jalovaara and Kreyenfeld (2020) for Finland (15% for men and 19% for women), and West Germany (13% for men and 16% for women). The UK estimates are very close to Guzzo (2014) whose paper suggested that in the USA, between 2006 and 2010, 13% of men aged 40 to 44 and 19% of women aged 41 to 49 had experienced MPF (Guzzo, 2014).

### Coresidential Partnership and Fertility Correlates of MPF

Table 2 shows the percentage of men and women who experience MPF according to selected coresidential partnership and fertility factors. Those with an earlier age at entry into parenthood and an earlier age at first coresidential partnership, and those who experienced multiple partnerships had higher reporting of MPF. For example, 59% of men and 50% of women who reported a first birth under the age of 19 experienced MPF as compared to 17% of men and 12% of women who had their first birth aged 25–29.

**Table 2:** Percentage experiencing MPF by age 42 according to select partnership and fertility factors

		Men				Women			
		Childless	No MPF	MPF	*Observations*	Childless	No MPF	MPF	*Observations*
Age at first birth^a^	19 and under	NA	41%	59%	*54*	NA	50%	50%	*219*
	20–24	NA	65%	35%	*624*	NA	63%	37%	*1048*
	25–29	NA	82%	17%	*958*	NA	88%	12%	*1147*
	30+	NA	96%	4%	*1518*	NA	96%	4%	*1340*
Age at first coresidential partnership	No partnership	92%	7%	1%	*278*	70%	27%	3%	*216*
	19 and under	13%	63%	24%	*336*	11%	61%	28%	*814*
	20–24	16%	70%	14%	*1553*	14%	72%	14%	*2061*
	25 and over	26%	65%	9%	*2078*	23%	66%	11%	*1552*
Total coresidential partnerships	No partnership	92%	7%	1%	*278*	70%	27%	3%	*216*
	One	19%	75%	7%	*2412*	14%	77%	9%	*2622*
	Two	24%	58%	19%	*1063*	19%	59%	23%	*1269*
	Three	24%	51%	25%	*362*	22%	48%	31%	*397*
	Four or more	33%	43%	23%	*132*	24%	39%	38%	*140*
Coresidential partnership dissolution	No partnership	82%	16%	2%	*278*	70%	27%	3%	*216*
	1 partnership no dissolution	14%	79%	7%^b^	*2070*	12%	81%	7%^b^	*2138*
	1 dissolution	28%	56%	16%	*1231*	20%	60%	20%	*1523*
	2+ dissolutions	29%	49%	22%	*667*	22%	47%	30%	*766*
Parity^a^	Two	NA	89%	11%	*1563*	NA	87%	13%	*1923*
	Three	NA	69%	32%	*584*	NA	64%	36%	*701*
	Four or more	NA	43%	57%	*231*	NA	44%	56%	*329*
Number of children reported in first coresidential partnership^c^	0	57%	39%	5%	*1856*	47%	47%	6%	*1865*
	1	NA	68%	31%	*712*	NA	59%	40%	*775*
	2	NA	90%	10%	*1193*	NA	87%	13%	*1470*
	3+	NA	88%	12%	*448*	NA	87%	13%	*517*
Total Observations^d^		1113	2671	487	4271	900	3067	687	4654
		(26%)	(62%)	(12%)	(100%)	(19%)	(66%)	(15%)	(100%)

Men and women born in Britain in 1970

^a^Those  who were childless were excluded

^b^For these individuals a birth was recorded outside of a coresidential partnership prior to the reporting of a first coresidential partnership

^c^Those who had all children outside a coresidential partnership were excluded

^d^Differences between total sample size and sample size for each partnership and fertility group were due to missing data or excluded data within the partnership and fertility groups

Amongst those who had two coresidential partnerships by age 42, 19% of men and 23% of women had experienced MPF, as compared to 25% of men and 31% of women who experienced three coresidential partnerships. The prevalence of MPF was particularly high amongst those who had just one birth in their first coresidential partnership: 31% of men and 40% of women. In comparison, 10% of men and 13% of women who had two births in their first coresidential partnership experienced MPF. Additionally, the average age at MPF, defined as the date of birth of a child with a new partner, was 30.3 years for men and 28.4 years for women, in comparison the average age at second birth for those who did not experience MPF was 32.7 years for men and 31.1 years for women.

### Educational Differentials in Multi-partner Fertility

Education has consistently been found to be a key driver in reducing socioeconomic disadvantage, improving health and wellbeing, and promoting health equity (Hahn & Truman, 2015). We therefore chose education to highlight SES differences in MPF prevalence, and used two measures of education to demonstrate this; firstly cohort members own education and secondly parental education. Parental education was chosen given it provides a good indication of the socioeconomic situation of the respondent when they were growing up. This provides a different measure than the respondent’s own level of education and is not affected by reverse causation; the respondent’s own education could potentially be the outcome of demographic events in early adulthood. For instance, having a teen birth might lead someone to stop pursuing education.

Table 3 shows the percentage of UK men and women experiencing MPF according to their own educational qualifications as reported at age 30. Table 4 shows the percentages experiencing MPF according to their parent’s highest level of education (reported when the cohort member was aged 5). This measure was derived from the highest level of education of either the mother or father, and for those with only one parent it is this level that is reported. The categories of education used in both tables are: no educational qualifications, CSE/GCSE/secondary level qualifications, and advanced or degree level qualifications. These cross-tabulations show bivariate relationships and not adjusted for other factors—we note the potential confounding effect of parent’s education attainment on respondent’s own educational attainment.

**Table 3:** Distribution of MPF estimated at age 42 for men and women by own education reported at age 30

	Men^a^					Women				
	No MPF		MPF			No MPF		MPF		
	Childless	Children with one partner	Children with two partners	Children with three or more partners	Observations	Childless	Children with one partner	Children with two partners	Children with or more partners	Observations
No qualifications	22.2%	57.9%	14.2%	5.6%	*1222*	15.2%	56.3%	18.1%	10.3%	*1064*
CSE/GCSE/secondary	24.7%	62.1%	11.0%	2.2%	*1222*	16.6%	65.6%	14.2%	3.7%	*1447*
A level and degree	27.4%	66.3%	5.4%	1.0%	*1547*	22.7%	66.3%	8.6%	2.5%	*1853*
Total observations	996	2493	392	110	3991	822	2775	557	210	4364
	(25.0%)	(62.5%)	(9.8%)	(2.8%)	(100%)	(18.8%)	(63.6%)	(12.8%)	(4.8%)	(100%)

^a^The difference in total observations between Tables 3 and 4 was due to missing data. For Table 3 those who reported MPF status but did not report education at age 30 were treated as missing, and for Table 4 those who reported MPF status but did report information on parental education at age 5 were treated at missing

**Table 4:** Distribution of MPF estimated at age 42 for men and women by highest education qualification of the cohort members’ parents reported at age 5

	Men^a^					Women				
	No MPF		MPF			No MPF		MPF		
	Childless	Children with one partner	Children with two partners	Children with three or more partners	Observations	Childless	Children with one partner	Children with two partners	Children with or more partners	Observations
No qualifications	24.2%	58.4%	12.8%	4.7%	*1093*	17.2%	59.8%	15.6%	7.4%	*1246*
CSE/GCSE/secondary	24.9%	63.9%	9.7%	1.5%	*1128*	18.8%	66.3%	11.4%	3.6%	*1274*
A level and degree	26.6%	66.8%	5.4%	1.1%	*938*	21.2%	67.4%	9.9%	1.6%	*993*
Total observations	796	1985	300	78	3159	665	2257	437	154	3513
	(25.2%)	(62.8%)	(9.5%)	(2.5%)	(100%)	(18.9%)	(64.2%)	(12.4%)	(4.4%)	(100%)

^a^The difference in total observations between Tables 3 and 4 was due to missing data. For Table 3 those who reported MPF status but did not report education at age 30 were treated as missing, and for Table 4 those who reported MPF status but did report information on parental education at age 5 were treated at missing

The descriptive results suggested that for both genders, those with lower educational attainment and whose parents had lower education attainment had higher reporting of having children with two or more different partners (MPF), compared to those with higher educational attainment. The proportion having children with at least three different partners was more than three times higher amongst men and women with no educational qualifications (men 6%; women 10%), as compared with those with advanced or degree level qualifications (men 1%; women 3%) (Table 3). Those with advanced and degree level qualifications were more likely to have remained childless (men 27%; women 23%), compared to those with no qualification (men 22%; women 15%) (Table 3).

The educational gradient in MPF remained when we focussed on the parental education attainment of the cohort members. For both genders, cohort members whose parents had no educational qualifications had higher reporting of children with two partners (MPF) (men 13%; women 16%), compared to those whose parents had a higher educational qualifications (men 5%; women 10%) (Table 4). The proportion having children with at least three different partners was more than three times higher amongst men and women whose parents had no educational qualifications (men 5%; women 7%), as compared with those whose parents had advanced or degree level qualifications (men 1%; women 2%). Those whose parents had advanced or degree level qualifications had higher reporting of remaining childless (men 27%; women 21%) compared to those whose parents had no qualifications (men 24%; women 17%) (Table 4).

In Supplementary Materials Tables 1 and 2, we additionally present MPF estimates by education but only for parents of parity two or higher. Restricting the sample to those of parity two or higher further widened the educational differentials in the prevalence of MPF.

## Discussion

This paper produced the first estimates of MPF for the UK, demonstrating how an indirect method can be successfully applied to birth cohort data. The paper provided a first insight into SES differences in MPF in the UK according to parental and cohort members own educational attainment levels. Depending on the assumptions used, amongst those born in Britain in 1970, between 12 and 14% of men and 15 and 18% of women experienced MPF by age 42.

These estimates are similar to those published using data for similarly aged adults in the USA and Finland. Our estimates were slightly higher than those from Norway, but lower than those from East-Germany and Sweden. Factors we hypothesise to be likely to contribute to MPF in the UK, such as high rates of teenage childbearing, high levels of partnership instability, and a significant proportion of higher order births (Berrington & Diamond, 2000; Carr & Springer, 2010; Holley et al., 2006; Schoen, 2020; Shearer et al., 2002), may not in fact result in levels of MPF that are particularly high by international standard. Future comparative research could identify the importance of these different drivers of MPF and examine whether relatively high rates of childlessness in the UK (ONS, 2022) are acting to reduce overall levels of MPF. As previously noted by Andersson (2021) and Jalovaara et al. (2022), MPF is associated with larger completed family sizes; and we found that around one third of men and women who had three children had experienced MPF, as compared to just over half of those with four children. Our research also showed that, in most cases, MPF occurred with just two different partners, supporting the conclusion that MPF is now most common amongst those who have a child with one partner and then separate, subsequently forming another partnership and then having additional children.

However, caution must be used when comparing MPF estimates, given the broad range of data sources, the geographical location of the data, the measurement approaches and populations studied. It is also important to consider contextual factors, specifically national differences in re-partnering behaviour and a country’s attitudes towards divorce and separation, and post-separation child custody arrangements, as these factors will be likely to influence the prevalence of MPF (Andersson, 2021; Gałęzewska et al., 2017). Further research should continue to build on work such as Thomson et al., 2021 paper and additionally include estimates of MPF from the UK into cross-national comparisons to understand whether these cross-national similarities and differences are genuine (i.e., not due to measurement differences), and if they are, explore what are the drivers of MPF in each country.

This paper identified significant differences in the likelihood of experiencing MPF according to individual’s own educational qualifications and the educational qualifications of their parents. Descriptive results demonstrated that cohort members who had no qualifications, or whose parents had no qualifications had higher levels of MPF compared to cohort members with advanced or degree level qualifications, or whose parents had advanced or degree level qualifications. Future research is required using multivariate analyses to account for educational differences in the demographic events that we have shown to be associated with MPF e.g. early childbearing, partnership dissolution and re-partnering. Our results go some way to supporting McLanahan’s (2004) ‘diverging destinies’ thesis: those with higher education attainment are more likely to lead stable family lives, whereas those with lower educational attainment experienced greater family instability and complexity. The results presented here demonstrated the potential use of MPF estimates and the need for further research to explore both determinants of MPF and the potential social, demographic and health effects of experiencing MPF.

### Strengths and Limitations

There are several strengths of this paper. Firstly, the BCS70 is a large British cohort study that despite attrition has retained a large sample. The fact that data collection started at birth and has continued across the life course provides the opportunity to explore repeated measures of coresidential partnership and fertility histories. However, MPF estimates should be interpreted with caution. We used an indirect approach for estimating MPF, and a caveat of this approach is that we were forced to make assumptions about fertility patterns and childbearing outside of coresidential partnerships. It is also important to note that an indirect approach may create more uncertainty in future MPF estimates if the number of non-coresidential childbearing partnerships continue to rise, as we will be forced to make an increasingly greater number of assumptions about childbearing outside of coresidential partnerships.

Whilst a direct approach to estimating MPF is preferable, such data are often unavailable, not least because of the questionnaire space required to include these questions. The approach detailed in this paper represents a method that allows for high quality datasets such as the BCS70 to be utilised to examine this important topic, when direct methods are not available. Robust conclusions about the comparability of direct and indirect measurements of MPF cannot be drawn from this data and can only be proven in a setting where both methods can be applied within the same sample. There is value in future work exploring if this comparison can be made in alternative datasets.

Researchers should be mindful of how family complexity is likely to relate to non-response bias. Individuals with lower education, lower parental education and unstable family biographies are more likely to drop out of longitudinal surveys and have more unstable partnership and fertility trajectories (Boertien, 2020; McLanahan, 2004; Mostafa & Wiggins, 2015; Stannard et al., 2019), therefore based on these issues we could be underestimating the prevalence of MPF. Finally, even though we were able to utilise childbearing and coresidential partnership histories collected prospectively at multiple time points, there might still be some recall errors especially in the retrospective reporting of coresidential partnership events between survey waves. Finally, we note that, although the BCS70 cohort is representative of children born in the UK in 1970, the current UK population is much more ethnically diverse than that represented in this data. Therefore, the findings reported here refer to a population of mainly white ethnicity.

## Conclusion

This paper was the first to estimate MPF prevalence in the UK and explore SES differences. This is an important addition to family and fertility literature given that partnership and fertility trends in the UK might lead us to expect high levels of MPF, but this had yet to be quantified. This paper has discussed the methods and data required to estimate MPF using an indirect approach that allows for high quality datasets such as the BCS70 to be utilised to examine this important topic when direct methods are not available. We have established that amongst a cohort of people born in 1970, MPF is a common family formation in the UK, but that levels are not particularly high compared with other Western countries. However, there are significant difference in MPF prevalence by own and parental educational attainment levels.

## Supplementary Information

Below is the link to the electronic supplementary material.

Supplementary file1 (DOCX 19 KB)
