# Supplementary material for: Educational Gradient of Multi-partner Fertility: First Estimates for the UK
Source: Eur J Popul. 2024 Jun 26;40(1):22. doi: 10.1007/s10680-024-09708-4 (PMC11208377; doi:10.1007/s10680-024-09708-4)
Supplement: Supplementary file 1 — Supplementary file1 (DOCX 19 KB) [file 10680_2024_9708_MOESM1_ESM.docx]

**Supplementary Materials**

**Supplementary Materials 1: Cleaning Partnership Histories Within BCS70**

The steps taken when repairing partnership history cases were as followed:

1. There were 17 participants who had no data from adulthood prior to sweep 6 (age 30) on partnerships although they had reported a separation/divorce. The CLS had tried to repair these cases using dummy variables; however, they did not include partnership start and end months, so all 17 participants had to be dropped.
2. Zero duration partnership – 141 partnerships were zero in duration (i.e., the start and end date were the same); of these partnerships 115 stated the partnership started the same month as their last interview date (these partnerships were retained). The 16 partnerships with zero duration and where the start date did not match the date of last interview were dropped.
3. Missing century month data. Overall, all the partnerships retained in the partnership histories included a start month. However, 298 partnerships did not have an end month but had stated they had separated. Because we required accurate partnership months to estimate Multi-Partner Fertility (MPF) and cannot repair these cases accurately these participants were dropped from the sample.
4. Partnership still intact. Those who had not separated at the last survey (age 42) need an ‘end month’. Therefore, we have altered partnerships which were still intact by indicating the partnerships ended at 1370 month. 1370 month was chosen because this is after the age 42 sweep of data collection.
5. 104 partnership end dates overlapped with the start date of the next relationship. For these cases the end date of the previous relationship was replaced with the start date of the next relationship.
6. 190 cases appear to be cohabitation with a previous spouse. For these cases we derive MPF separately to ensure that MPF is accurate.

|  | Men | | | | Women | | | |
| --- | --- | --- | --- | --- | --- | --- | --- | --- |
|  | 2 or more children with one partner | Children with two partners | Children with three or more partners | Sample | 2 or more children with one partner | Children with two partners | Children with or more partners | Sample |
| No qualifications | 478 (63.9%) | 191 (25.5%) | 78 (10.4%) | 747 | 453 (57.1%) | 220 (27.7%) | 120 (15.1%) | 793 |
| CSE/GCSE/secondary | 564 (75.5%) | 152 (20.4%) | 31 (4.2%) | 747 | 741 (73.1%) | 217 (21.4%) | 56 (5.5%) | 1014 |
| A level and degree | 777 (88.1%) | 84 (9.5%) | 20 (2.3%) | 882 | 932 (81.5%) | 167 (14.6%) | 44 (3.9%) | 1143 |
| *Total* |  |  |  | *2376* |  |  |  | *2950* |

**Supplementary Materials Table 1. The percentage distribution of MPF estimated at age 42 for men and women by own education reported at age 30, for parents of parity two or higher.**

**Supplementary Materials Table 2. The percentage distribution of MPF estimated at age 42 for men and women by highest education qualification of the cohort members parents reported at age 5, for parents of parity two or higher.**

|  | Men | | | | Women | | | |
| --- | --- | --- | --- | --- | --- | --- | --- | --- |
|  | 2 or more children with one partner | Children with two partners | Children with three or more partners | Sample | 2 or more children with one partner | Children with two partners | Children with or more partners | Sample |
| No qualifications | 647 (69.6%) | 214 (23.0%) | 68 (7.3%) | 930 | 767 (66.2%) | 272 (23.5%) | 120 (10.3%) | 1159 |
| CSE/GCSE/secondary | 471 (80.4%) | 92 (15.7%) | 23 (3.9%) | 586 | 572 (76.3%) | 138 (18.4%) | 40 (5.3%) | 750 |
| A level and degree | 436 (88.4%) | 45 (9.1%) | 11 (2.2%) | 493 | 481 (81.9%) | 89 (15.2%) | 17 (2.9%) | 587 |
| *Total* |  |  |  | *2009* |  |  |  | *2496* |
